# Nondestructive Estimation of Muscle Contributions to STS Training with Different Loadings Based on Wearable Sensor System

**DOI:** 10.3390/s18040971

**Published:** 2018-03-25

**Authors:** Kun Liu, Yong Liu, Jianchao Yan, Zhenyuan Sun

**Affiliations:** School of Mechanical Science and Engineering, Jilin University, Changchun 130025, China; kunliu@jlu.edu.cn (K.L.); liuyong16@mails.jlu.edu.cn (Y.L.); zysun17@mails.jlu.edu.cn (Z.S.)

**Keywords:** nondestructive, joint moment, partial weight loading, muscle contributions, sit-to-stand training

## Abstract

Partial body weight support or loading sit-to-stand (STS) rehabilitation can be useful for persons with lower limb dysfunction to achieve movement again based on the internal residual muscle force and external assistance. To explicate how the muscles contribute to the kinetics and kinematics of STS performance by non-invasive in vitro detection and to nondestructively estimate the muscle contributions to STS training with different loadings, a wearable sensor system was developed with ground reaction force (GRF) platforms, motion capture inertial sensors and electromyography (EMG) sensors. To estimate the internal moments of hip, knee and ankle joints and quantify the contributions of individual muscle and gravity to STS movement, the inverse dynamics analysis on a simplified STS biomechanical model with external loading is proposed. The functional roles of the lower limb individual muscles (rectus femoris (RF), gluteus maximus (GM), vastus lateralis (VL), tibialis anterior (TA) and gastrocnemius (GAST)) during STS motion and the mechanism of the muscles’ synergies to perform STS-specific subtasks were analyzed. The muscle contributions to the biomechanical STS subtasks of vertical propulsion, anteroposterior (AP) braking and propulsion for body balance in the sagittal plane were quantified by experimental studies with EMG, kinematic and kinetic data.

## 1. Introduction

Sit-to-stand (STS) movement is a common, but critical action that almost every independent person performs every day, but millions of people have difficulty in STS motion due to neurological pathologies globally [1]. STS movement is also a complex dynamic task that requires the regulation of lower limb muscles to drive the human body and maintain the dynamic balance while performing subtasks, such as leaning forward movement of the HAT (head-arms-trunk segments) in the chair, vertical stretching movement of the whole body and balancing movement of the whole body off the chair [2,3,4,5]. Patients with a decrease in leg muscle function experience difficulties in achieving STS movement, but improvement may occur with proper training [6,7,8,9]. STS movement requires coordination of several muscle groups to guarantee human balance while achieving the task. However, for the elderly and dependent people who have lost part of the lower limb functionality, the activity becomes tiring and cannot be accomplished without the help of external assistance [10]. To restore muscle strength and improve STS movement coordination, repetitive STS training with appropriate external support is very important for the patients with lower limb motion impairments. Consequently, assistant systems, such as exoskeleton/orthosis or a partial body weight support (PBWS) rehabilitation robot, can be used to provide and control external assistance [11,12,13,14].

A greater understanding of the biomechanics of STS and muscle contributions to STS has important implications for rehabilitation training. It is valuable for clinicians to make a targeted, effective and efficient physical therapy plan in individual STS rehabilitation training with motor disability. In common STS movement, the primary mechanism to regulate joint moments is muscle force generation. Muscles accelerate body segments and generate ground reaction forces that alter joint moments about the segments’ center-of-mass (COM) to restore and maintain dynamic stability. In addition, gravity contributes to whole-body angular momentum through its contribution to the ground reaction force (GRF) [15]. During STS rehabilitation training, since certain muscles of the lower limb are weak due to neurological pathologies or surgery injury, the patients need external assistant forces (EAF), such as PBWS force on the shoulder or assistant torque on the knee joint. Accordingly the patient is driven to stand up by lower limb muscles and EAF cooperatively. However, because the individual situation varies, it is hard to quantitatively evaluate the muscles’ rehabilitation phase and to accurately estimate the muscle contributions to dynamic body support without intrusive observation and measurement. Few previous studies have quantified which muscles primarily contributed to whole-body angular momentum of STS. Identification of muscles’ responsibilities and contributions in STS motion has important implications for diagnosis and treatment of balance and movement disorders and the design of effective rehabilitation therapies that target specific muscle groups. Gravity acts throughout the segment, but can be located at the COM for calculation purposes. The gravity contributes to joint reaction forces, and negative joint moments with the moment arms from each segment’s COM to the corresponding joint consumed the joint moments that are offered by muscle synergies. There have been many studies on the biomechanics of STS, and many papers have reported findings related to electromyographic (EMG) activity during the action [16,17,18]. The applications of computational biomechanics for orthopedic treatment and rehabilitation have attracted much attention to provide better quality of life for patients. Especially a greater understanding of the biomechanics and muscle activity of STS movement has important implications to design safe, effective and efficient rehabilitation training guidelines for individuals with motor disability [19,20,21]. Therefore, non-invasive estimation of joint moments, joint forces and muscle contributions to body support with loading for STS training using a wearable sensor system is very important for STS rehabilitation.

McGowan et al. [22] offered a method to estimate lower limb muscles’ contributions to body support and forward propulsion. However, they just analyzed independent effects of weight and mass on plantar flexor activity during walking, not STS movement. Neptune et al. [23] analyzed the muscle contributions to whole-body sagittal plane angular momentum during walking. These results may provide insight into balance and movement disorders and provide a basis for developing locomotor therapies that target specific muscle groups, but there was no practical significance for STS rehabilitation. Wang et al. predicted the joint moments using a neural network model of muscle activations from EMG signals [24], but the method was used to calculate the elbow joint moments, not the lower limb joint moments from the EMG signals of ten flexor and extensor muscles. Thomas Stieglitz et al. presented a noninvasive measurement of torque development in the rat foot, but it is based on electrode implantation in the plantar/dorsiflexion and medial/lateral rotation plane. In a sense, it is not really a noninvasive measurement [25]. Furukawa et al. [26] introduced a newly-developed biosignal-based vertical weight support system, which was composed of pneumatic artificial muscles (PAMs) and an EMG measurement device. It proved that external assistance force can be varied based on measured muscle activities and can be used to instruct STS rehabilitation training. Bonnet et al. [27] investigated the possibility of estimating hip and knee joint angles using a single inertial measurement unit, but they did not pay attention to the rehabilitation of lower limb muscles. Zlatko et al. analyzed and compared kinematics, kinetics and EMG patterns of STS transfer [28] and proved they can be used for STS training. Tatsuya analyzed an EMG-based predictive control model for physical human-robot interaction, but it was not suitable for STS training [29]. Nicole et al. analyzed muscle contribution and coordination during stair ascent using a 26-camera optoelectronic motion capture system. The insightful studies provided a detailed understanding of the biomechanics during stair ascent at the joint level, but it’ is not a real-time and wearable method for analyzing joint moments [30].

In this paper, the differences of muscle excitation intensity in different phases during STS are identified. The purpose of the study is to identify the functional roles of the lower limb individual muscles during STS movement and the mechanisms by which muscles work together to perform STS-specific subtasks. The muscle contributions to STS motion at the individual muscle level were analyzed by experimental studies with EMG data, kinematic and kinetic data. The STS kinetics and kinematics were non-invasively estimated and analyzed using developed wearable inertial sensors. The patterns of five groups of the lower limb muscles during standing up were characterized by processed EMG to examine the relationship between the muscle activities and STS kinetics. Furthermore, to identify individual muscle contributions to the biomechanical STS subtasks of vertical propulsion, anteroposterior (AP) braking and propulsion and to analyze how individual muscles, EAF and gravity contribute to STS motion and body balance in the sagittal plane, a partial-muscle-actuated STS movement was generated with different loadings on the HAT of the subject. Based on inverse dynamics analysis of the STS biomechanical model, we estimated hip, knee and ankle joint moments from accelerations, angular velocities and angles measured on the lower limb segments (HAT, thigh and shank) using inertial sensors. The results showed insight into the movement coordination of STS and had implications for the ongoing development and testing of more effective STS training techniques in the clinic.

## 2. Methods

**Figure 1:**
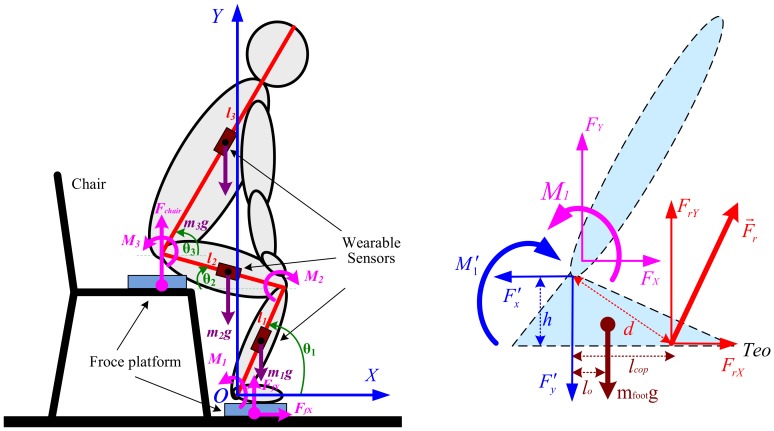
Link segment model of dynamic sit-to-stand (STS) biomechanical model.

Compared to horizontal walking during which the COM of the body is predominantly propelled, STS motion requires an individual to propel the COM significantly more vertically and less horizontally. In addition, it is obvious that dynamic balance is more difficult to maintain after the body left the seat (seat-off). These differences suggest that STS motion likely requires altered muscle contributions relative to different phases before and after seat-off. To estimate the complex relationships between muscle excitation and resultant force, the muscle contributions to accelerate joints and segments in the STS biomechanical task should be analyzed based on the STS dynamic biomechanical model as shown in Figure 1. The subjects rise from sitting in an essentially sagittal symmetric manner; therefore, a two-dimensional three-link segmental model is used in the analysis of the STS task under the assumption of bilateral symmetry in the sagittal plane.

In most tasks involving kinetic measurements of the STS subject, a direct measurement of internal moments acting on lower limb joints was not feasible for PBWS rehabilitation robot users. A dynamic STS biomechanical model of a trainee consisting of the HAT, thighs, shanks and feet is needed to estimate and analyze joint moments with external GRFs, loading forces and kinetic and kinematic parameters (inertial effects). In addition, initial feet position in the STS biomechanical model directly affects the distance that HAT must move forward in STS motion. It was proven that the amplitude of displacement and velocity at the hip could be significantly optimized when the feet are behind the knee in the AP direction [31,32]. Thus, the STS movement is divided into four phases [33,34]: in the first phase, the subject in the sitting position with feet under the knees bends forward the HAT. In the second phase, the subject lifts off the chair. In the third phase, the subject extends the knee joint and the whole body stretches. Then, in the final phase, the subject is full upright in the stand-up position. Therefore, the human body can be represented by three different parts, shank-foot, thigh and HAT, and can be modeled for the STS movements as a triple inverted pendulum model in the sagittal plane articulated around the ankle, knee and hip joints. In our presented work [35], we had non-invasively estimated the joint moments using the developed wearable sensor system and analyzed the kinematic and kinetic profiles underlying the STS movement. As a further analysis of STS using wearable sensors, we recalculated the joint moments *Mi* (*i* = 1, 2, 3) based on the inertial and static effects of STS motion as follows.

Hip joint moment is shown with Equations (1)–(3):

(1) $${\vec{M}}_{3-INERTIA}=\frac{1}{m}\cdot (\frac{1}{2}\cdot J_{3}\cdot {\vec{\alpha }}_{3}+{\vec{r}}_{33}\times (\frac{1}{2}\cdot m_{3}\cdot {\vec{a}}_{3}))$$

(2) $${\vec{M}}_{3-STATIC}=\frac{1}{m}\cdot ({\vec{r}}_{33}\times (\frac{1}{2}\cdot m_{3}\cdot \vec{g}))$$

(3) $${\vec{M}}_{3}={\vec{M}}_{3-INERTIA}+{\vec{M}}_{3-STATIC}$$

Knee joint moment is shown with Equations (4)–(6):

(4) $${\vec{M}}_{2-INERTIA}=\frac{1}{m}\cdot (-\frac{1}{2}\cdot J_{3}\cdot {\vec{\alpha }}_{3}-J_{2}\cdot {\vec{\alpha }}_{2}-{\vec{r}}_{23}\times (\frac{1}{2}\cdot m_{3}\cdot {\vec{a}}_{3})-{\vec{r}}_{22}\times (\frac{1}{2}\cdot m_{2}\cdot {\vec{a}}_{2}))$$

(5) $${\vec{M}}_{2-STATIC}=\frac{1}{m}\cdot (-{\vec{r}}_{23}\times (\frac{1}{2}\cdot m_{3}\cdot \vec{g})-{\vec{r}}_{22}\times (m_{2}\cdot \vec{g}))$$

(6) $${\vec{M}}_{2}={\vec{M}}_{2-INERTIA}+{\vec{M}}_{2-STATIC}$$

Ankle joint moment is shown with Equations (7)–(9):

(7) $${\vec{M}}_{1-INERTIA}=\frac{1}{m}\cdot (\frac{1}{2}\cdot J_{3}\cdot {\vec{\alpha }}_{3}+J_{2}\cdot {\vec{\alpha }}_{2}+J_{1}\cdot {\vec{\alpha }}_{1}+{\vec{r}}_{13}\times (\frac{1}{2}\cdot m_{3}\cdot {\vec{a}}_{3})+{\vec{r}}_{12}\times (m_{2}\cdot {\vec{a}}_{2})+{\vec{r}}_{11}\times (m_{1}\cdot {\vec{a}}_{1}))$$

(8) $${\vec{M}}_{1-STATIC}=\frac{1}{m}\cdot ({\vec{r}}_{13}\times (\frac{1}{2}\cdot m_{3}\cdot \vec{g})+{\vec{r}}_{12}\times (m_{2}\cdot \vec{g})+{\vec{r}}_{11}\times (m_{1}\cdot \vec{g}))$$

(9) $${\vec{M}}_{1}={\vec{M}}_{1-INERTIA}+{\vec{M}}_{1-STATIC}$$

where ${\vec{M}}_{i}$ is the joint moment vector about joint *i*; $J_{j}$ the moment of inertia of segment *j* about the center of mass; ${\vec{\alpha }}_{j}$ the angular acceleration vector of segment *j* about the center of mass; ${\vec{r}}_{ij}$ the position vector from joint *i* to the center of gravity of segment *j*; $m_{j}$ the mass of segment *j*; m the whole body mass with loading on the HAT; ${\vec{a}}_{j}$ the acceleration vector of the center of gravity of segment *j*.

To quantify the causal relationships between the lower limb muscle excitation inputs and the resulting STS performance, the musculoskeletal modeling with explicit equations for the neuromuscular and musculoskeletal system dynamics should be utilized. Previous studies have used simulations to identify individual muscle contributions to tasks such as walking [36] and wheelchair propulsion [37]. Recently, Lin et al. [38] performed a simulation analysis to determine the contributions of five muscle groups to body support, forward propulsion and balance control during stair ascent by analyzing whole-body COM accelerations. The insightful studies in [30] provided a detailed understanding of the biomechanics of stair ascent. It was obvious that the five lower limb muscles (rectus femoris (RF), gluteus maximus (GM), vastus lateralis (VL), tibialis anterior (TA) and gastrocnemius (GAST)) among the analyzed fifteen muscles made the main contributions to the vertical propulsion. Therefore, to nondestructively identify the functional roles of the lower limb individual muscles during STS training movement, our research focused on the five lower limb muscles’ contributions to the biomechanical STS subtasks of vertical propulsion, AP braking and propulsion in the sagittal plane. Meanwhile, to develop a wearable succedaneum of the bulky camera optoelectronic motion capture system for detailed understanding of how individual muscles, EAF and gravity contribute to STS motion and body balance, a wearable sensor system was developed.

The mass and dimension of each segment of the subject were estimated based on the average current Chinese male inertia parameters of body segments according to Chinese national standards, as shown in Table 1. The moment of inertia of each segment was estimated based on the height and the total mass of the subject [39] and shown in Table 1. All segments were assumed to be rigid, and the STS movement was performed only in the sagittal plane.

## 3. Experiment

A sensor system composed of two force plates, inertial sensor modules (IMUs) and EMG electrodes was developed. To measure the kinematic parameters of the segments for calculating joint moments according to Equations (1)–(9), three customized IMUs (wearable sensor JY-901B, 1.1 × 1.1 × 0.5 inches with battery and Bluetooth communication) were attached on the position of COM of the shank, thigh and HAT with Velcro, as shown in Figure 1. A microcontroller (Arduino UNO) was used to capture accelerations and angular velocities from the IMUs at a 100-Hz sampling rate, store data and communicate with a PC in real time. The two force plates were developed with pressure sensors (YZC-1B), and the forces were sampled at a 100-Hz rate using the microcontroller (Arduino UNO). Force Plate A was fixed on the chair to measure the vertical chair reaction force (VCRF) before seat-off. Force Plate B was placed under subjects’ feet to measure the vertical ground reaction force (VGRF). During the initial calibration, the force plates were positioned horizontally with calibration errors of 0.74% and 0.68% of the measured vertical forces. To relate each IMU’s orientation to the corresponding segment’s orientation, a sensor-to-segment calibration procedure is performed as presented in [40]. The X-axis of each IMU was repeatedly adjusted to coincide with the axial direction of its corresponding segment in the segment coordinate frames based on the recommendations of the International Society of Biomechanics [41]. The wearable sensor system has been verified in our previous research work [35] to be available for non-invasive estimation of joint moments and analysis of STS kinematics.

**Figure 2:**
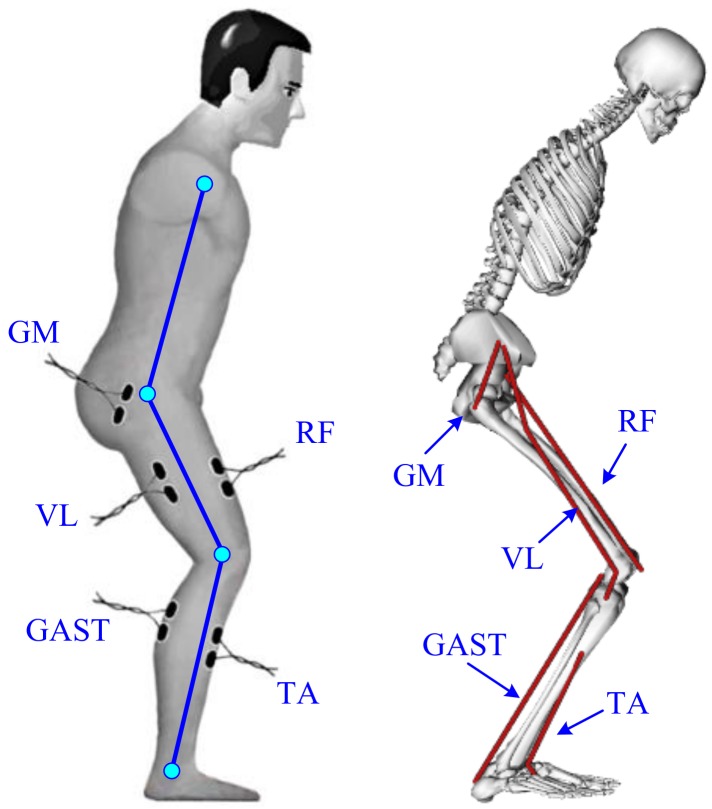
Schematic presentation of the EMG electrodes’ placement and muscles in the sagittal plane. GM, gluteus maximus; VL, vastus lateralis; GAST, gastrocnemius; RF, rectus femoris; TA, tibialis anterior.

**Figure 3:**
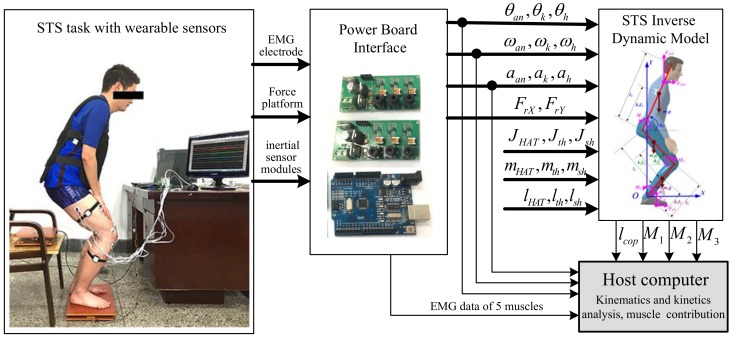
Schematic diagram of the proposed method.

EMG signals from five lower limb muscles of the right leg, RF, GM, VL, TA and GAST, were monitored by bipolar surface electrodes (Meditrace 0.11-m self-adhesive Ag/AgCl electrodes). The electrode positions are indicated in Figure 2. The proximal electrode was positioned above either the bulkiest part or the middle of the muscle belly with a 0.01-m standard interelectrode distance according to [42]. At the electrode locations, the skin was shaved, cleaned with alcohol and lightly abrased to further reduce skin resistance. Electrical silence in all five muscles was achieved prior to data collection. The EMG signals were processed by means of two customized self-developed 3-channel amplifiers as shown in Figure 3 with a band-pass filter from 10 Hz–500 Hz. The signal was amplified (1 K) and converted from the analog-to-digital form via an analog-to-digital interface board (PCI 1713U) at a sampling frequency of 1000 Hz. To procure a relative impression of the degree of activity, all EMG signals were full-wave rectified and low-pass filtered.

Ten healthy male subjects (age 26 ± 2 years, height 1.74 ± 0.05 m, weight 66 ± 6.5 kg) without any known musculoskeletal or neurological dysfunction participated in this study and received informed consent. The experimental protocol was approved by the Human Ethical Review Committee of Jilin University. After familiarization and practice, the subjects reported no serious impediment of the developed sensor system. Then, each subject performed three STS trials as a task and five STS tasks with different loadings (0 kg, 5 kg, 10 kg, 15 kg and 20 kg) on the HAT at a self-selected preferred speed and movement pattern without any support. In each initial sitting posture of the STS trials, the feet position were located behind the knee in the anteroposterior direction with self-selected appropriate distance of about half the length of the foot. Therefore, 150 trials (3 trials × 5 groups of tasks per subject × 10 subjects) were achieved. Although a task of three STS trials was performed by one subject, the STS time and the amount of captured data of each trial could not be absolutely the same. Three events provided reference points for kinematic data: movement onset (when the angular velocity of the COM of HAT being greater than 0.01 rad/s), seat-off (zero VCRF) and movement end (when the angular velocity of the COM of thigh was less than 0.01 rad/s or VCRF approximately equaled the subject’s weight). The three events enabled the action to be divided into two phases: a pre-stretch phase (movement onset to seat-off) and a stretch phase (seat-off to movement end). Therefore, the time of seat-off (zero VCRF) was designated as the referenced standard point (RSP) within whole STS to synchronize the kinematic, kinetic and EMG data derived from the three STS trials of a task to the same percentage metric, then the ensemble averages of a task were obtained. The kinematic data, VGRF, VCRF and the calculated joint moments were normalized to the subject’s height and weight, then were used to analyze the muscle contributions to body support in different-loading STS training compared with the five groups of EMG data captured from RF, GM, VL, TA and GAST.

## 4. Results

**Figure 4:**
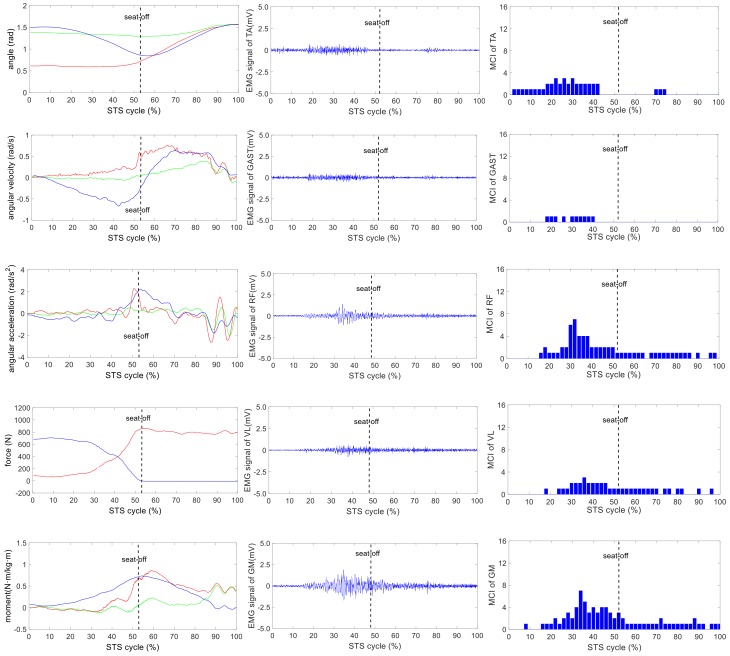
The analysis result of a typical STS task with no loadings: **the left column** shows the hip, knee and ankle joint angles and moments (green, red and blue lines, correspondingly), angular velocities and angular accelerations of the HAT, thigh and shank (green, red and blue lines, correspondingly), and vertical chair reaction force (VCRF) and vertical ground reaction force (VGRF) (blue and red lines, correspondingly). **The middle column** shows the surface EMG signals of the five lower limb muscles (TA, GAST, RF, VL and GM). **The third column** shows the muscle contribution indexes (MCI) of the five lower limb muscles (TA, GAST, RF, VL and GM).

**Figure 5:**
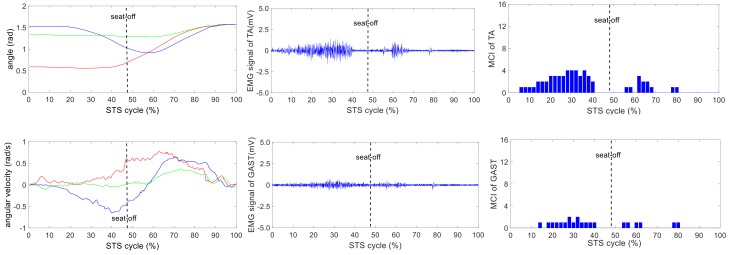

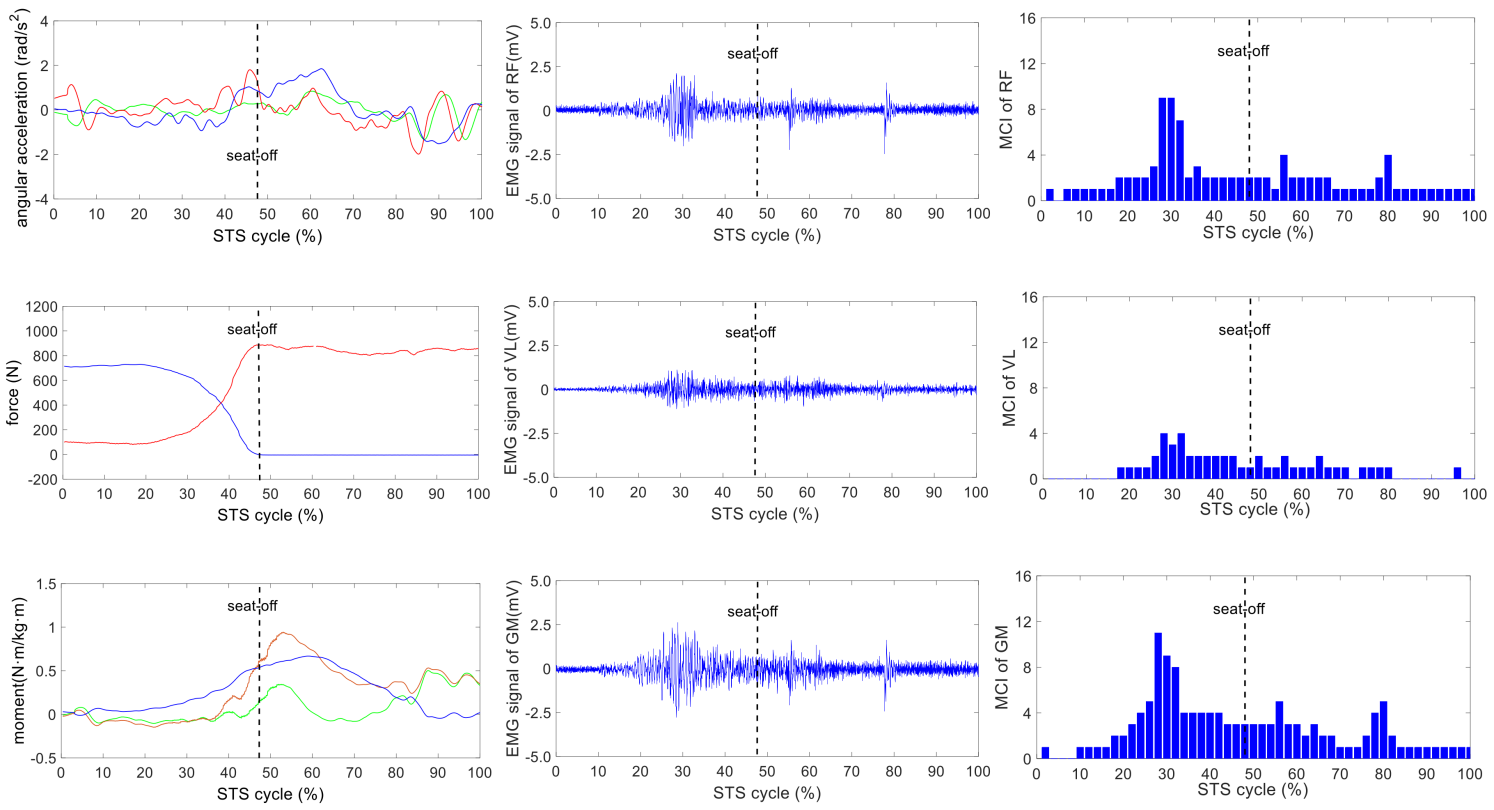
The analysis result of a typical STS task with 5-kg loadings: **the left column** shows the hip, knee and ankle joint angles and moments (green, red and blue lines, correspondingly), angular velocities and angular accelerations of the HAT, thigh and shank (green, red and blue lines, correspondingly) and VCRF and VGRF (blue and red lines, correspondingly). **The middle column** shows the surface EMG signals of the five lower limb muscles (TA, GAST, RF, VL and GM). **The third column** shows the muscle contribution indexes (MCI) of the five lower limb muscles (TA, GAST, RF, VL and GM).

**Figure 6:**
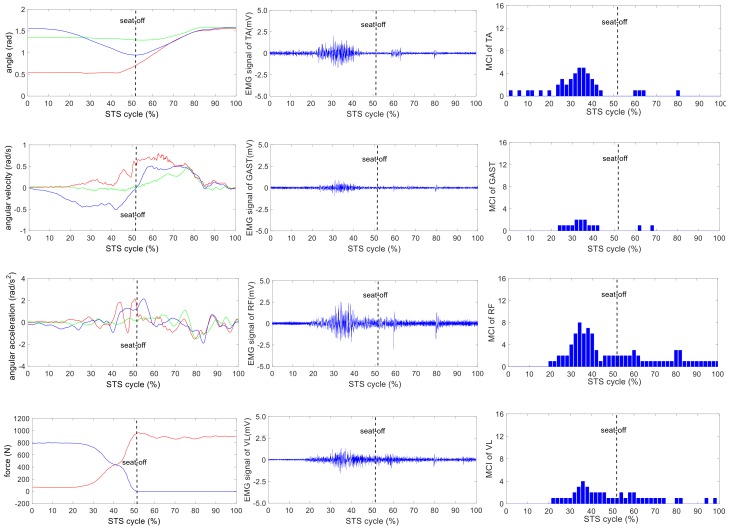

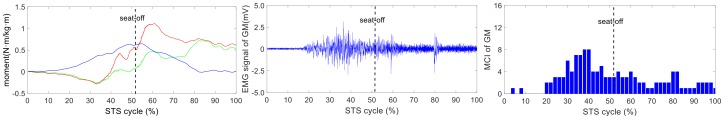
The analysis result of a typical STS task with 10-kg loadings: **the left column** shows the hip, knee and ankle joint angles and moments (green, red and blue lines, correspondingly), angular velocities and angular accelerations of the HAT, thigh and shank (green, red and blue lines, correspondingly) and VCRF and VGRF (blue and red lines, correspondingly). **The middle column** shows the surface EMG signals of the five lower limb muscles (TA, GAST, RF, VL and GM). **The third column** shows the muscle contribution indexes (MCI) of the five lower limb muscles (TA, GAST, RF, VL and GM).

**Figure 7:**
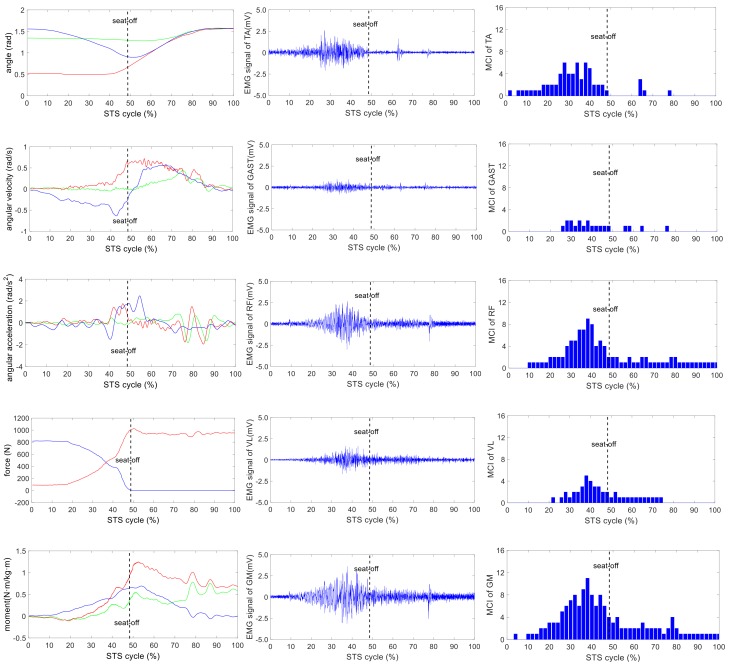
The analysis result of a typical STS task with 15-kg loadings: **the left column** shows the hip, knee and ankle joint angles and moments (green, red and blue lines, correspondingly), angular velocities and angular accelerations of the HAT, thigh and shank (green, red and blue lines, correspondingly) and VCRF and VGRF (blue and red lines, correspondingly). **The middle column** shows the surface EMG signals of the five lower limb muscles (TA, GAST, RF, VL and GM). **The third column** shows the muscle contribution indexes (MCI) of the five lower limb muscles (TA, GAST, RF, VL and GM).

**Figure 8:**
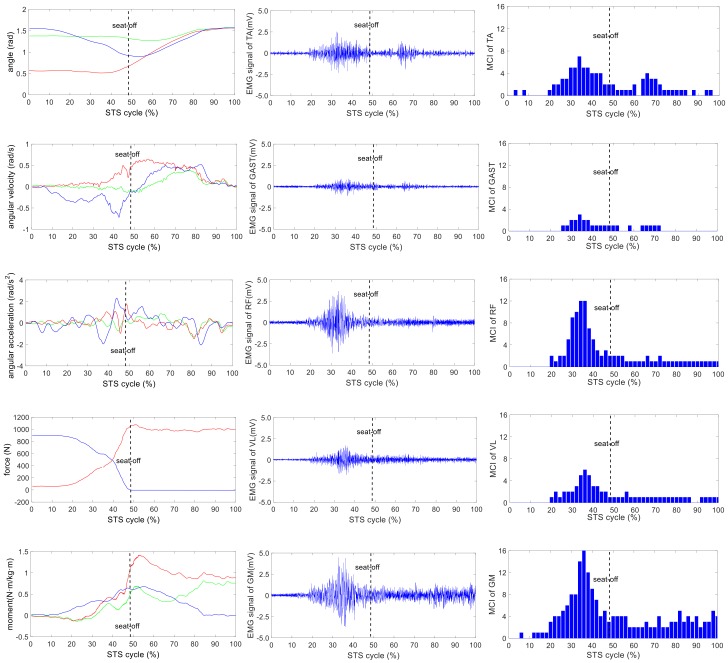
The analysis result of a typical STS task with 20-kg loadings: **the left column** shows the hip, knee and ankle joint angles and moments (green, red and blue lines, correspondingly), angular velocities and angular accelerations of the HAT, thigh and shank (green, red and blue lines, correspondingly) and VCRF and VGRF (blue and red lines, correspondingly). **The middle column** shows the surface EMG signals of the five lower limb muscles (TA, GAST, RF, VL and GM). **The third column** shows the muscle contribution indexes (MCI) of the five lower limb muscles (TA, GAST, RF, VL and GM).

The wearable sensor system has been verified in our earlier research work [35]. It was available for non-invasive estimation of joint moments and analysis of STS kinematics. All signals captured by the developed sensor system were off-line processed by MATLAB. A typical group of the ensemble averages of the synchronized kinetic and kinematic parameters and EMG signals derived from five different loading STS tasks performed by one healthy subject is shown in Figure 4, Figure 5, Figure 6, Figure 7 and Figure 8. In each figure, the first column shows the calculated hip, knee and ankle joint angles and moments (green, red and blue lines, correspondingly), angular velocities and angular accelerations of the HAT, thigh and shank (green, red and blue lines, correspondingly) and VCRF and VGRF (blue and red lines, correspondingly). The second column shows the surface EMG signals of the five lower limb muscles (TA, GAST, RF, VL and GM). The third column shows the bar graph of the analysis of the muscle contribution index (MCI) based on the surface EMG signal after further calculation.

Voluntary muscle activity detection from EMG signals can be problematic due to spurious involuntary spikes produced by physiological and extrinsic/accidental origins [43]. Accurate detection of surface EMG signals for the lower limb muscle activities is important to understand how muscles made the contributions to STS motion. However, the EMG signal distortion is unavoidable with the removal of the spike contamination, thus impeding reliable measurement of weak EMG signals. The study in [44] demonstrated the usefulness of the integrated profile of EMG for muscle activity detection using surface EMG signals in the presence of spurious background spike contamination. The integrated profile method was used to determinate the onset of muscle contraction and contribution to STS motion without removing spurious background spikes from raw surface EMG signals. In this paper, as further processing of the integrated profile of EMG, MCI is defined to quantitatively indicate the five lower limb muscles’ contributions to the biomechanical STS subtasks of vertical propulsion, AP braking and propulsion in the sagittal plane. The MCI of each muscle was calculated from the ensemble averages of the synchronized integrated profile of EMG of the three STS trials in the same task and was quantified by 50 integers in a whole STS cycle by percentage metric.

## 5. Discussion

As indicated in [35], faster and more fluent STS movements resulted in better accuracy of the kinetic and kinematic analysis of STS using the developed inertial sensor system. Since it was more difficult to firmly fix the IMUs on the soft human body segments than on a rigid body without any slight movement, the systematic error was predictable, but inevitable. Especially in lower speed STS motion, the long duration of skin motion artifact due to impact loading and muscle activation and body-sway motion in non-fluent STS trials would inevitably contaminate the measured original angular velocities, accelerations and the raw EMG signals.

**Figure 9:**
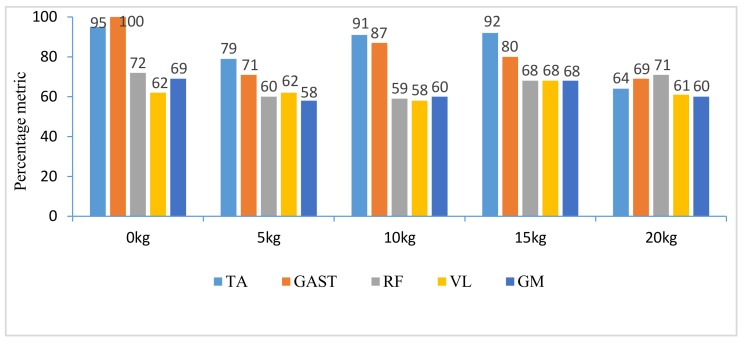
Percentage metric of each muscle’s accumulated contribution before seat-off in different tasks.

The difference of the five muscles was due to their relative contributions to the horizontal and vertical GRFs and joint moments resulting in different kinematics of STS motions with different loadings. Referring to the joint angles of the hip, knee and ankle in the first column of Figure 4, Figure 5, Figure 6, Figure 7 and Figure 8, it is suggested that the HAT took action first among the three segments of the subject. When the HAT was still swinging forward before the hip joint angle reached the maximum, the knee joint angle had begun to increase since seat-off; meanwhile, the HAT continued to swing forward into the stretch phase (after seat-off), then the ankle joint began to increase in the terminal stretch phase. Compared with the EMG signals, MCIs and the kinetic and kinematic parameters in Figure 4, Figure 5, Figure 6, Figure 7 and Figure 8, the highest levels of the five muscles’ excitation intensities (indicated by maximum MCI) all occurred before seat-off. Meanwhile, as shown in Figure 9, the percentages of each muscle’s accumulated MCI before seat-off were all no less than 58% of the total contributions to whole STS motion, which suggests that the muscles’ contributions to the whole STS motion primarily concentrated before seat-off compared to those after seat-off. However the max knee joint moment appeared after seat-off, and the HAT still swung forward after seat-off into the stretch phase. It is, therefore, quite clear that the muscles’ co-contractions were recruited ahead of the stretch subtask before seat-off. The superimposition before seat-off could be intended as a synergic action of muscles for controlling weight bearing as static joint moments, then the muscle synergies continued to increase until the inertial joint moments were adequate to promote vertical propulsion, anteroposterior (AP) braking and propulsion for performing a balanced body support of STS motion.

**Figure 10:**
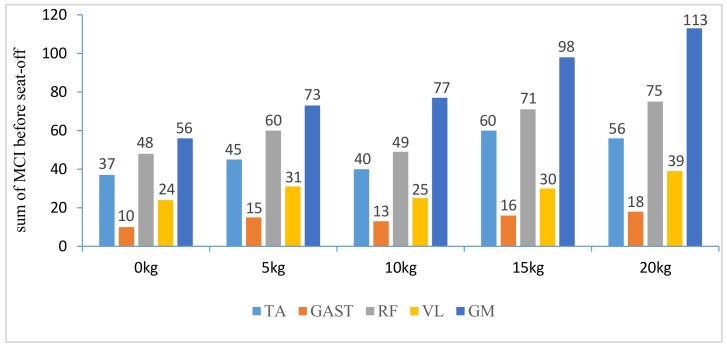
The sum of each muscle’s MCI before seat-off in different tasks.

**Figure 11:**
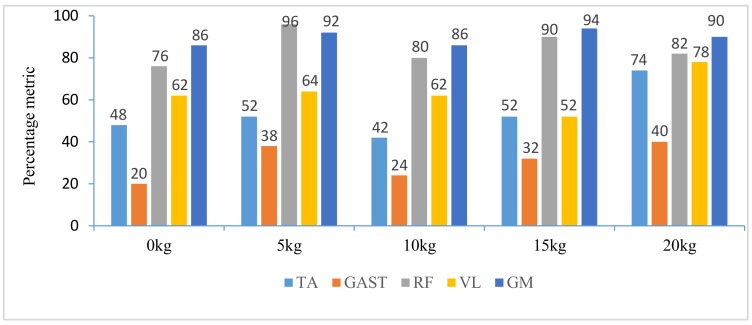
Percentage metric of each muscle’s contraction duration in whole STS cycle of different loading tasks.

In addition, with regard to the sum of MCI of each muscle before seat-off in different loading tasks in Figure 10, the absolute accumulated MCI of GAST before seat-off was the least among the five muscles in any task (no more than 18 in the 20-kg task, minimum MCI = 10 in the 0-kg task), and as shown in Figure 4, Figure 5, Figure 6, Figure 7 and Figure 8, the MCI of GAST was even weaker or not detected after seat-off. This suggested that GAST made the least contribution to STS motion among the five muscles in any loading task, but almost all of its contributions were made before seat-off (100% in the 0-kg task in Figure 10). It is, therefore, quite clear that GAST primarily contributed to the preparation of a stable stretch phase by holding the shank before seat-off and mainly contributed to the ankle joint extension and holding after seat-off. Compared the MCIs of TA and GAST in Figure 4, Figure 5, Figure 6, Figure 7 and Figure 8, TA had the same characteristic as GAST to contribute synergy for holding the shank and ankle joint extension before seat-off, but it was obvious that TA contracted earlier (almost from the beginning) and contributed more synergy in duration and intensity than GAST. Compared with the onset times of the five muscles in each loading task, GAST and VL contracted relatively the latest of the five muscles (almost after 20% of the STS cycle). This once again suggested that the primary contributions of GAST (including VL here) were to vertical STS propulsion preparation and balance holding of the lower limb, but not to the swing of HAT before seat-off. Besides, with regard to the onset time and MCI of each muscle in Figure 4, Figure 5, Figure 6, Figure 7 and Figure 8, the muscles’ activities in the stretch phase were recognized as typical activations of RF muscle as knee extensors, GM muscle as hip extensors and TA muscle as ankle extensors. Comparing the joint angles and moments with the corresponding MCIs of GM, TA and RF in the same task in Figure 4, Figure 5, Figure 6, Figure 7 and Figure 8, the high correlation of the onset time and amplitude suggested that the GM, TA and RF were the primary contributors to forward propulsion of HAT from the beginning, then all five muscles co-contracted to apply sufficient synergies for vertical propulsion until the subject seated off. Obviously, as shown in Figure 11, the GM contributed the most among the five muscles in duration and intensity to accelerate HAT forwards in the pre-stretch phase and keep stable hip joint extension in the stretch phase in any loading task. For example, in Figure 10, before seat-off in each task, the accumulated MCI of GM was 56, 73, 77, 98 and 113, respectively, any of which was the biggest of the five muscles in the same loading task. It is obvious in Figure 10 that, with the addition of loadings, the muscles’ contributions indicated by MCI also increased. This fully illustrates the causal relationship between muscle force and loadings. For example, in the successful STS task with the 20-kg loading, as shown in Figure 8, all of the MCIs of the five muscles indicated the maximum contributions compared with other loading tasks. On the contrary, this also suggested that deficits in the neuromuscular control of the five selected muscles could adversely influence balance recovery, which may be important targets in rehabilitation to improve balance recovery performance. As the MCIs of VL, RF and GM in each task show, their synergic action after seat-off was still obvious. This suggests that the synergies were presented to assist knee and hip extensions and develop muscle tension for weight and external loading acceptance in the stretch phase, meanwhile providing the majority of braking accelerations for the balance recovery in the terminal stretch phase before movement end.

Kinetics analysis in the stretch phase: To control STS balance, the antagonistic muscles spanning the hip, knee and ankle joints distributed power from the leg to the remaining body segments. This also suggested that the uniarticular hip and knee extensors (GM and VL) and ankle dorsiflexors (TA) generated backward angular momentum while the ankle plantar flexors (GAS) generated forward momentum; therefore, the whole body began vertical propulsion with enough kinetic energy into the stretch phase, then the muscles’ co-contractions obviously reduced with less MCIs after seat-off. That RF and VL overlapped their activities in the early stretch phase just after seat-off (refer to the third column of Figure 4, Figure 5, Figure 6, Figure 7 and Figure 8) was likely synergizing for modulating rapid knee flexion, although in less than 10% of the whole STS cycle and with low levels of MCI.

In particular, there are still some uncommon and variable activities of the TA, RF and GM indicated by significant MCIs in the middle and terminal stretch phases before movement end (in the third column of Figure 4, Figure 5, Figure 6, Figure 7 and Figure 8), probably because with the stretching of the body segments and the vertical propulsion of the COM after seat-off, the primary work of lower limb muscles had converted to support body balance. RF and VL supposedly contributed to knee flexion and patella stabilization in the middle and terminal stretch phase; RF and GM participated in hip stabilization; and TA and GAST contributed to ankle flexion and stabilization. According to the consultancies with subjects, as evidence, the deficiency of sufficient stability of the self-made force plate indeed caused an instantaneous stretch balance adjustment in the terminal stretch phase. Especially in the tasks with more loadings (15 kg and 20 kg), TA, RF and GM should instantaneously recruit obvious muscle synergies for stretch balance. Compared with the MCIs of TA, RF and GM in the stretch phases of the five loading tasks in Figure 4, Figure 5, Figure 6, Figure 7 and Figure 8, the MCIs of the three muscles became higher with more obvious fluctuation as the loadings increased, so this confirmed that more loadings would induce more muscle activities and co-contractions of TA, RF and GM for balance control in the stretch phase.

The results and discussion could provide insight into STS balance and movement disorders and offer a reference for developing locomotor therapies that target specific muscle groups; meanwhile, it may provide further rationale for developing targeted STS rehabilitation robot control strategies to address patient-specific deficits in STS training. Although the selected lower limb muscles’ relative contributions to STS motion were compared and analyzed based on the muscle excitation intensities and durations and the kinetics and kinematics of STS motion, outcomes on muscle co-contraction and absolute contributions of more lower limb muscles should be confirmed and supported by further analysis. In addition, differences in designated loadings and self-selected STS speeds across subjects could introduce variability in muscle onset-offset times and co-contraction. Thus, STS speed and loadings could be acknowledged as a limitation of the study.

## 6. Conclusions

Quantification of lower limb muscles’ co-contraction contributing to STS propulsion movement with different loadings was analyzed based on a self-developed wearable sensor system. How the muscles generate, absorb and/or transfer mechanical power between body segments to accelerate the whole-body COM was identified by quantitative assessment of five lower limb muscles’ contributions during able-bodied STS motion, in terms of variability of onset-offset muscular activation, excitation intensity indicated by MCI and the percentage occurrence of EMG signals and kinetic and kinematic information derived from the wearable sensor system. The results would contribute to further understanding of more muscles’ contributions and coordination during STS movement and provide additional insight for the development of effective, targeted rehabilitation programs aimed at improving an individual’s ability to STS rehabilitation training and useful in the clinical context for designing future STS or gait studies. In the future, partial body weight support or more specified loading would be tested on patients in rehabilitation by more motion models, including assisted standing motion with hands or weight support rehabilitation robots. Finally, the presented method of nondestructive estimation of muscle contributions to STS motion based on the wearable sensor system is expected to be applied to weight support or loading rehabilitation robot control or evaluation of STS rehabilitation therapy.

## Figures and Tables

**Table 1:** Average inertia parameters of body segments of current Chinese male adults according to Chinese national standards [39]. HAT, head-arms-trunk.

Segments (Definition)	Segment Length/ Height (%)	Segment Mass/ Whole Body Mass (%)	Center of Mass/ Segment Length Distal	Moment of Inertia (kg·m²)
Foot (lateral malleolus/ head metatarsal)	14.77	3.6	0.5	0.0044
Shank (femoral condyles/ medial malleolus)	23.86	10.6	0.567	0.0385
Thigh (greater trochanter/ femoral condyles)	28.13	22.7	0.567	0.1978
HAT (greater trochanter/ glenohumeral joint)	50.17	63.1	0.374	0.9180
